# Ohio Lunatic Asylum

**Published:** 1847-02

**Authors:** 


### OHIO LUNATIC ASYLUM.

We thank the officers of this institution, for continuing to send us their Annual Report to the Legislature. That now before us, for the year 1846, is number eight. Most of its predecessors we have already noticed. The following extract contains matter worthy of the attention of every State in the Union:

"The directors are much gratified in being able to announce that this great enterprise of benevolence may now be considered as completed. *The work is done.* Its conception and execution belong to the people of Ohio. The work is emphatically theirs. In all the agitations and mutations of parties—in all the monetary revulsions which have occurred since it was commenced, its progress has never been for a moment improperly delayed. Not a solitary voice of dissension has been heard upon the subject. Every year has witnessed the spectacle of a whole people, through their representatives, coming up as one man to lay their united offerings upon the shrine they had consecrated to the most touching and pitiable of human woes."

We copy the following paragraphs, for the purpose of rousing to effort those States in the West, which have not yet provided Asylums for their insane. To say nothing of the comforts they secure to that class of patients, the number of cures effected in the establishment at Columbus, is truly encouraging, and should prompt to action elsewhere.

"The Institution went into operation in the month of November, 1838. Since that time there have been in it 866 patients; 461 males, and 405 femaels; 247 pay patients, 649 supported by the State; 358 have been discharged cured, 92 have died; 420 were "*recent cases*," (of less than a year's duration when the patient was received,) 446 were old cases, (of more than a year's duration.) Of the recent cases discharged, 90.59-100 per cent. (or 289) were cured—of the old cases 27 per cent. (or 69.) In addition to this, a great number of those incurable have been much improved in their condition.

"During the past year 175 patients have been admitted, 88 males, and 87 females. Of these, 101 were 'recent cases,' 74 were old cases; 71 have been discharged 'cured,' 18 have died. In the recent cases discharged, 95·38-100 per cent. were cured—in the old cases 20·93-100 per cent. A number are still improving, with fair prospects of recovery. These results compare favorably with those in the best institutions, both in this country and abroad."

In running over the statistics of this Report, we observe a number of interesting facts, but our limits do not permit an extensive transcript.

The following table, embracing all the patients, 866, admitted from the opening of the establishment in 1839, is instructive as to the time of life, at which insanity commences in this country, and its relative prevalence in the two sexes:

*Showing the age at which Insanity commenced.*

| | Males. | Females. | Total. |
|---|---|---|---|
| Under twenty years | 38 | 47 | 85 |
| From twenty to thirty years | 216 | 160 | 376 |
| " thirty to forty years | 108 | 106 | 214 |
| " forty to fifty years | 66 | 60 | 126 |
| " fifty to sixty years | 24 | 27 | 51 |
| " sixty to seventy years | 9 | 5 | 14 |

It would seem, that the seasons exert some influence on this disease. Of three hundred and forty-seven recoveries, fifty-three took place in winter; seventy-nine in spring; ninety-one in summer, and one hundred and twenty-four in autumn. The number of the latter season, contrasts too strikingly with the numbers which respectively represent the other seasons, to be accidental. If we take winter and spring together, and compare them with summer and autumn, we have 132:215. From which it would appear, that hot weather is more favorable to the cure of insanity than cold.

The statistics of intemperance and insanity are worthy of notice. They run through eight years, and if we divide the whole time into two equal periods, we have for the former 11·18 per cent.—for the latter 5·30 per cent., of cases ascribed to intemperate drinking. But this will appear more obviously by the following table:

FIRST PERIOD.

| | | |
|---|---|---|
| A. D. 1839 | 14·50 | per cent. |
| 1840 | 11·00 | " |
| 1841 | 8·25 | " |
| 1842 | 10·75 | " |

SECOND PERIOD.

| | | |
|---|---|---|
| A. D. 1843 | 6·25 | per cent. |
| 1844 | 5·75 | " |
| 1845 | 6·00 | " |
| 1846 | 3·50 | " |

Now to what should we ascribe this diminution since 1842? this reduction to one-fourth, from 1839 to 1846? Undoubtedly to the influence of the great Temperance Reform, which began to spread over the West since 1840. The epidemic cholera of 1832–'34 gave a great impulse to whiskey and brandy drinking, of which the fruits were fully developed in 1839. Soon after that date, an era of comparative sobriety began, and its beneficial effects were presented to our admiration in 1846, by a reduction in the percentage of three-fourths! Let those who are wont to sneer at the efforts to stay the tide of intemperance, look at this result, and apply it to the whole country.

Of four hundred and sixty-three patients whose occupations are given, we observe that two only are physicians, while six are lawyers. As the number of the former in the State of Ohio far exceeds that of the latter, it follows that the practice of law favors the production of insanity more than that of medicine.

The treatment of the insane in this public charity, which does honor to the State of Ohio, is largely of a moral character. Thus, a reading-room, extensively furnished with newspapers and magazines, has been provided, and several anniversaries have been observed by the patients. The 4th of July is celebrated; the annual State election is held within, as without the walls of the Asylum, and every practicable method adopted for exercising the faculties and interesting the feelings of its inmates—the whole of which is conducted by the indefatigable Dr. Awl, of whom the Directors say: "Much praise is due to the superintendent, under whose care the institution has grown up from infancy to its present magnitude. The system adverted to is the work of his hand."

We should not, however, do full justice to our noble profession, always foremost in works of benevolence, if we did not add that the name of S. Parsons, which stands at the head of the Board of Directors, is that of one of the ablest and most respectable physicians of the State.

